# Effects of oro-esophageal versus nasogastric feeding on dysphagia for ischemic stroke survivors: study protocol for a randomized controlled trial

**DOI:** 10.3389/fnut.2026.1830508

**Published:** 2026-04-29

**Authors:** Mingming Wang, Chenyue Xia, Yi Lu, Hongji Zeng, Hongyan Liu, Weijia Zhao, Liugen Wang, Xi Zeng, Ying Yang

**Affiliations:** 1 Department of Rehabilitation Medicine and TCM, The People’s Hospital of Suzhou New District, Suzhou, China; 2 Suzhou Hospital of Traditional Chinese Medicine, Nanjing University of Chinese Medicine, Suzhou, China; 3 School of Health Policy and Management, Peking Union Medical College, Chinese Academy of Medical Sciences, Beijing, China; 4 Department of Respiratory and Critical Care Medicine, The Second Medical Center, National Clinical Research Center for Geriatric Diseases, Chinese PLA General Hospital, Beijing, China; 5 Department of Medicinal Insurance, The Third Affiliated Hospital of Zhengzhou University, Zhengzhou, China; 6 Department of Rehabilitation Medicine, The First Affiliated Hospital of Zhengzhou University, Zhengzhou, China; 7 Dysphagia Research Institute, Zhengzhou University, Zhengzhou, China

**Keywords:** dysphagia, enteral nutrition, rehabilitation, stroke, swallowing disorder

## Abstract

**Background:**

Enteral nutrition is commonly practiced for ischemic stroke survivors with dysphagia. In Eastern Asia, nasogastric and oro-esophageal tubes are the mainstream options. However, there is a lack of rigorous clinical evidence on the effects of these two feeding methods on swallowing-related rehabilitation outcomes and clinical relevance.

**Objectives:**

This study is clinically oriented and aims to assess the effect of nasogastric versus oro-esophageal tube feeding on the degree and speed of dysphagia improvement, and aspiration symptoms.

**Methods:**

This multicenter randomized controlled trial will include 422 ischemic stroke patients with dysphagia who require tube feeding. Stratified randomization will be performed to assign participants 1:1 to the oro-esophageal or nasogastric groups. All participants will receive 15-days routine rehabilitation care and nasogastric or oro-esophageal feeding, according to their group assignment.

**Outcomes:**

The primary outcome is the dysphagia severity assessed using the Dysphagia Outcome and Severity Scale (DOSS). The secondary outcomes include time to improvement of one level from the baseline DOSS, time to oral intake, accumulation of secretions assessed using the Murray Secretion Scale, pharyngeal residue after swallowing assessed using the Yale Pharyngeal Residual Severity Rating Scale, and airway protection assessed using the Penetration-Aspiration Scale. Aspiration symptoms will be monitored for 6 weeks.

**Discussion:**

This study aims to provide evidence-based support for the comprehensive effects of tube feeding on swallowing-related rehabilitation outcomes.

**Clinical trial registration:**

ClinicalTrials.gov, identifier NCT07386834.

## 1 Introduction

Dysphagia affects 30–50% of ischemic stroke survivors (1). It significantly increases the risk of malnutrition, aspiration, and subsequent pneumonia (2). Enteral nutritional support is clinically practiced for patients requiring non-oral nutrition (2). In East Asia, public acceptance of gastrostomy is relatively low, and nasogastric tubes are the primary option (3). However, several studies have shown that nasogastric tubes may increase the risk of adverse outcomes, including reflux, pharyngeal discomfort, elevated muscle tone, and psychological problems (3–5). To address these issues, intermittent oro-esophageal tube feeding was first conceptualized in 1988 (6). This method involves oral insertion of tubes into the upper esophagus. The tube is placed before each meal and removed after feeding (3). Currently, this technique is mainly adopted in China, South Korea, and Japan.

With the progress of research, some teams have begun to focus on the effects of oro-esophageal versus nasogastric tubes on multiple rehabilitation outcomes, among which swallowing function is the most representative (7). Some scholars believe that oro-esophageal tubes can improve swallowing outcomes by stimulating the posterior pharyngeal wall, improving nutritional status, and promoting mental well-being (3, 7, 8). Our pilot study showed that oro-esophageal tubes significantly improved airway protection and oral intake limitation than nasogastric feeding (8). These conclusions are also supported by some clinical evidence (7). However, the following limitations have yet to be addressed: First, it remains unclear whether there is a difference in the speed of improving swallowing outcomes between oro-esophageal and nasogastric tubes. Obviously, significant improvement within the first few days of intervention has relatively great clinical significance. Second, it is difficult to quantify clinical significance in many studies in which outcomes were assessed using continuous scores. Third, most studies focus on general swallowing outcomes, while little is known about local functional status, such as the accumulation of secretions and pharyngeal residue after swallowing. Fourth, instrumental assessments are the gold standard for dysphagia, but have not been widely used (2). Most studies adopted screening tools or observation-based assessments (7). In addition, it is unknown whether the conclusions can be generalized to inpatients with ischemic stroke, which is the most common stroke subtype. Finally, the lack of follow-up for overt aspiration and aspiration pneumonia limits our understanding of the safety differences between oro-esophageal and nasogastric feeding.

To address these issues, this protocol plans to use Flexible Endoscopic Evaluation of Swallowing (FEES) and grade assessments to quantify swallowing function in ischemic stroke survivors (9). This study aims primarily to answer the following questions: First, is there clinical significance between the effects of oro-esophageal and nasogastric tubes on swallowing function? This includes the degree, speed, and practical differences in the outcomes. Second, are some key local swallowing functions affected? Finally, aspiration symptoms, including overt aspiration and aspiration pneumonia, will be followed for a period of time after the intervention ends to explore broader impacts.

## 2 Methods

### 2.1 Study design and patient and public involvement

**Figure 1:**
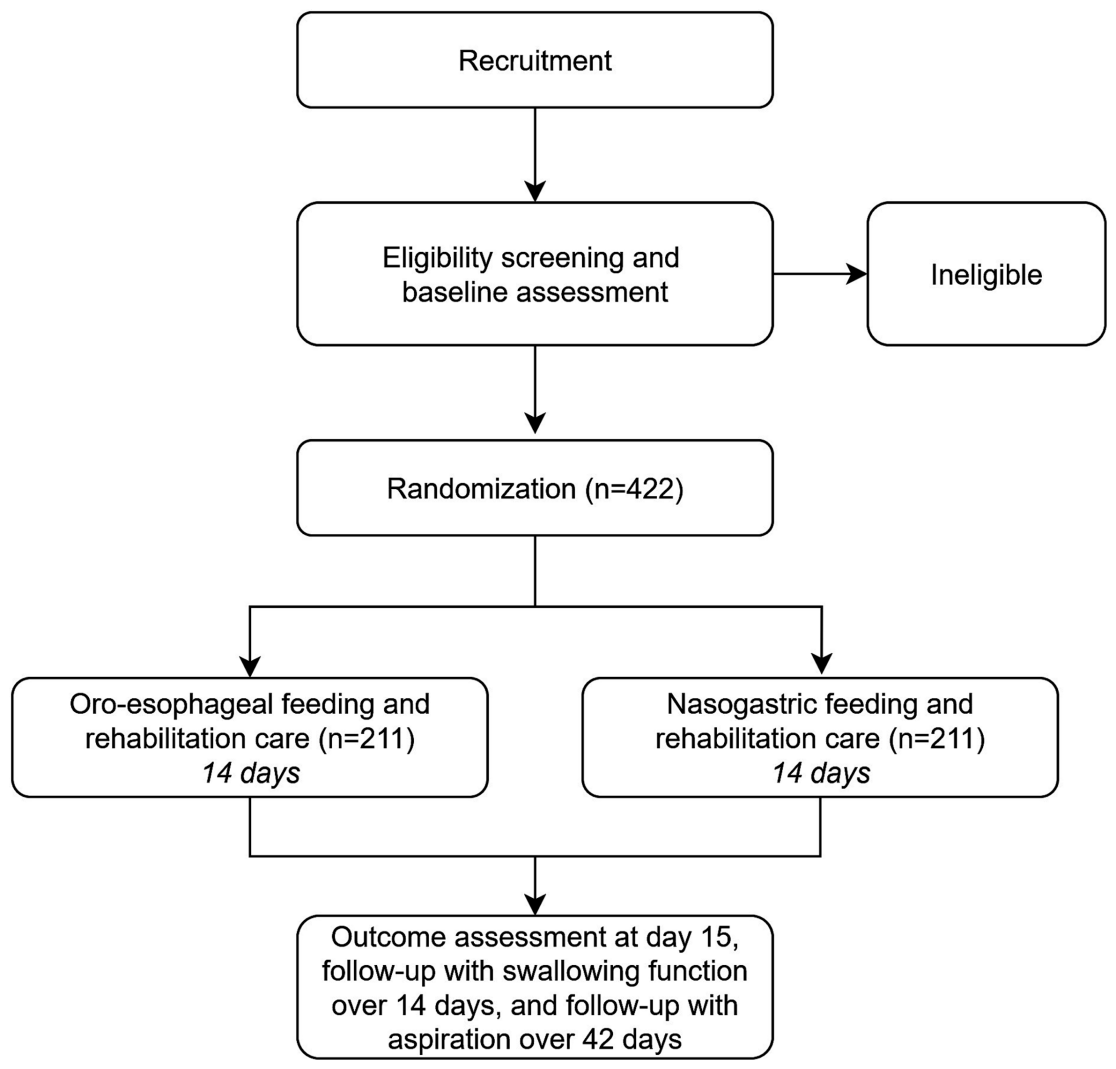
Trial design.

This randomized controlled study will include ischemic stroke survivors with dysphagia who require enteral nutrition support in rehabilitation departments. Participants will be randomized 1:1 into the oro-esophageal or nasogastric groups to receive enteral feeding accordingly for 14 consecutive days. The study design is shown in Figure 1. The current protocol is designed and reported according to the CONSORT and SPIRIT 2025 statements (10, 11), while referencing the TIDieR checklist (12). The public and patients had not been directly involved in the study design.

### 2.2 Participants

Participants will be recruited from six rehabilitation departments across five cities in China (Suzhou, Kaifeng, Xingtai, Harbin, and Zhengzhou). The inclusion criteria are as follows: (i) Age over 18 years; (ii) First-ever stroke; (iii) Ischemic stroke based on imaging data; (iv) Enteral nutritional support is required (13); (v) Stable vital signs; (vi) Clear consciousness; and (vii) Dysphagia Outcome and Severity Scale (DOSS) level 1–2 (14); (viii) Informed consent and willingness to comply with the study procedures. The exclusion criteria are as follows: (i) Dysphagia that might be caused by other etiologies or structural abnormalities of the swallowing process; (ii) Severe hepatic, renal or hematological diseases, or malignant tumors; (iii) Contraindications to tube feeding; (iv) Pregnancy or breast feeding; and (v) Anticipated discharge or transfer to other hospitals within 2 weeks.

### 2.3 Randomization and blinding

Randomization will be performed after obtaining informed consent and completion of baseline assessments, conducted separately at each trial site hospital by independent researchers using SAS version 9.4. Stratified randomization will be performed according to the DOSS levels to randomly assign participants in a 1:1 ratio to the oro-esophageal or nasogastric groups, with a block size of 4 or 6. Randomization information will be stored in the Clinical Research Methodology Key Unit and will remain inaccessible to irrelevant people.

The interventionists and participants will be unblinded, as the feeding method is identifiable. Some assessments will be scheduled during the intervention period, and these assessors will be unblinded. The assessors for day-15 assessments will be independent and blinded, as the feeding tube will be removed after the intervention period ends.

### 2.4 Intervention

The intervention period will last for 14 days. All participants will receive routine rehabilitation care, as shown in Table 1. These treatments adhere to local relevant guidelines (15, 16). Swallowing rehabilitation interventions will be arranged based on the Chinese Dysphagia Rehabilitation Management and Guidelines (Appendix S2) (17). There are no intergroup differences in the rehabilitation care across the trial site hospitals.

**Table 1:** A summary of the core standardized components, structure, and implementation of the intervention.

Intervention	Description	Timepoint and frequency
Risk factor control	Interventions are implemented for stroke-related risk factors, including blood pressure, blood lipid levels, blood glucose levels, tobacco and alcohol consumption, and physical activity. These interventions encompass lifestyle modifications (e.g., smoking and alcohol cessation, dietary adjustments) and pharmacological interventions (e.g., hypoglycemic agents, antihypertensive drugs).	Routine
Stroke recurrence prevention	Stroke recurrence prevention is a systematic intervention strategy aimed at reducing the risk of recurrent cerebrovascular events in patients with a history of stroke. Beyond risk factor control, pharmacological therapy is administered based on the etiological subtypes of stroke, such as aspirin, statins, and anticoagulants.	Routine
Health education	Health education is a critical component of stroke recurrence prevention that bridges the gap between clinical interventions and patient adherence, ensuring that patients and their caregivers fully understand and implement secondary prevention measures. It includes (1) Disease Knowledge Education, (2) Medication and Rehabilitation Compliance Education, (3) Lifestyle Intervention Guidance, (4) and (5) Psychological Adjustment Guidance	Day 1
Swallowing rehabilitation	All patients receive identical daily swallowing behavior rehabilitation (30–40 min/session, 6 days/week), including oral motor training, pharyngeal facilitation, swallowing maneuvers, and secretion management.	Monday to Saturday morning
Oral hygiene	Mouthwash and dental cleaning tools are used to help patients clean their oral cavity.	Every morning
Psychological intervention	Communication is conducted with patients and their caregivers to identify potential psychological issues. For those with suspected psychological problems, emotional stabilization is achieved through verbal comfort, peer-to-peer communication among patients, and meditation.	Routine
Other rehabilitation	For participants with functional impairments other than dysphagia, corresponding physical therapy and occupational therapy are arranged. As for swallowing function, distinctive interventions are provided based on the equipment available at each center, such as transcutaneous electrical stimulation, music therapy, and the Rood technique. For participants with cognitive impairment, routine cognitive training is provided once daily for approximately 30 min, including memory training, attention training, and executive function training conducted under the guidance of therapists.	After swallowing behavior rehabilitation
Nutritional support	To ensure full reproducibility and cross-center consistency, both feeding interventions follow fixed, protocolized implementation rules with identical nutritional targets, positioning, insertion/confirmation, feeding frequency, volume progression, and post-feeding care.	Routine

On the night before day 1, any prior enteral nutrition method will be discontinued. Starting from the first intervention day, the participants will receive enteral nutrition support based on their groups. Oro-esophageal feeding will be adopted for the oro-esophageal group. This silicone tube is 40 cm in length with an inner diameter of 5.4 mm, with some side holes at the tip and a syringe connector at the distal end, as shown in Appendix S1. During feeding, the participants will be instructed to be in a sitting or semi-sitting position. A trained nurse will slowly insert the tube into the upper esophagus via the corner of the mouth. Successful placement of the tube can be confirmed by the following conditions: (1) The nurse place the distal end of the tube under water, and no bubbles appear when the participant exhales; and (2) When the nurse gently rotates the tube, the resistance is normal and the participant does not cough. The initial feeding amount is 200 mL, which is increased by 50 mL per meal until reaching the participant’s daily dietary intake. Feeding will be performed 4–6 times daily, alternating between food and water. The feeding tube is removed after each feeding (3, 17). For the nasogastric group, successful tube placement can be confirmed by aspirating gastric juice with a syringe after the feeding tube is inserted. The nasogastric tube is secured to the user’s face, and feeding is administered in accordance with local nasogastric feeding guidelines (18). The nutritional standards are identical for both groups: daily caloric intake of 20–35 kcal/kg of body weight, with a carbohydrate-to-lipid ratio of 7:3 to 6:4. Daily protein demands are estimated using the 24-h urinary urea nitrogen (19).

### 2.5 Outcomes

**Figure 2:**
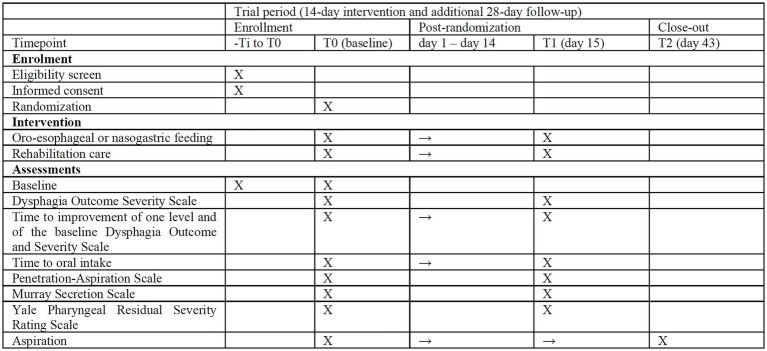
SPIRIT 2025 diagram of the schedule of enrollment, interventions, and assessments.

Several assessment timepoints are defined as follows: baseline (T0), after the intervention period ends at day 15 (T1), and follow-up at day 43 (T2). The SPIRIT participant timeline is shown in Figure 2 (10).

#### 2.5.1 Primary outcome

The DOSS will be used to assess dysphagia severity at T0 and T1 (14). This assessor-blinded assessment will be based on FEES (9), which has been recognized as one of the gold standards for dysphagia diagnosis (20–23). Participants will be instructed to swallow boluses of varying consistencies and volumes, and this procedure has been standardized and widely followed in previous studies (24). The DOSS is an ordinal seven-level scale, where a higher grade indicates more severe dysphagia (14). Among these grades, Level 1 and Level 2 are categorized as grades that require non-oral nutrition (25).

#### 2.5.2 Secondary outcomes

Based on the FEES at T0 and T1, (1) the Murray Secretion Scale (MSS), (2) the Yale Pharyngeal Residual Severity Rating Scale (YPR-SRS), and the Penetration-Aspiration Scale (PAS) will be used to assess the accumulation of secretions, pharyngeal residue after swallowing, and airway protection (26–28). The MSS, YPR-SRS, and PAS are ordinal four-, five-, and eight-level scales, respectively, with higher grades indicating more severe conditions. The MSS, YPR-SRS, and PAS are assessor-blinded. (4) The time to improvement of one level from the baseline DOSS will be recorded (14). A one-level increase in the DOSS is considered clinically significant as it indicates reduced need for non-oral feeding, lower risk of aspiration and pneumonia, earlier transition to oral intake, and improved functional independence. However, it is apparently impractical to conduct daily instrumental swallowing assessments. Therefore, a three-step decision-making strategy is designed to determine clinical improvement. First, researchers will conduct preliminary evaluations by observing the participants’ daily conditions, feeding, and swallowing training performance. Participants identified as having the potential for a one-level improvement will undergo a swallowing screen, which will be compared with the baseline results for definitive evaluations. Then, detailed communication will be conducted to obtain participants’ perception of the swallowing condition. Subsequently, researchers will decide whether to conduct an additional FEES to verify the degree of the DOSS improvement. This design is intended to minimize intra-individual heterogeneity in swallowing assessments and to incorporate patient performance, patient perceptions, caregiver observations, and instrumental assessments, with details shown in Appendix S3. (5) Time to oral intake will be recorded, defined as the first day, during the two-week intervention period, on which a participant meets level 3 or above on the Dysphagia Outcome and Severity Scale. (6) Aspiration symptoms will be monitored for 6 weeks. Participants will be instructed to record daily on a paper or electronic calendar the occurrence of overt aspiration, the materials causing the aspiration, and whether the aspiration is resolved. Overt aspiration is defined as coughing or discomfort triggered by the aspiration of food or water into the airway (29). Resolution of aspiration is defined as the disappearance of discomfort with behavioral steps, such as coughing or back patting. To address potential recall bias and underreporting of aspiration, some measures are taken, as shown in Appendix S4. To monitor silent aspiration, new-onset aspiration pneumonia will be tracked over a 6-week period (Appendix S4). After discharge, this study will accept aspiration pneumonia diagnosed by qualified physicians at any tertiary hospital, as long as the primary diagnosis is coded as ICD-10 J69.0 or the patient is explicitly diagnosed with aspiration pneumonia (30). The time to improvement of one level from the baseline DOSS, time to oral intake, and aspiration symptoms are open-label assessments.

### 2.6 Adverse events

Any adverse event will be monitored throughout the intervention period. Both researchers and participants will be instructed to report any unexpected events. Based on previous studies (8), some typical potential adverse events have been predefined and communicated to researchers and participants, including reflux, vomiting, abdominal distension, throat or pharyngeal discomfort, fever, and aspiration pneumonia. All adverse events will be continuously monitored, graded, and subjected to etiological analyses. Serious adverse events will be reported to the data-monitoring committee for third-party oversight.

### 2.7 Sample size

Previous studies have lacked assessments using the gold standard. Therefore, we conducted a preliminary study to determine the sample size. In this study, 48 ischemic stroke patients were randomly assigned in a 1:1 ratio to the OE and NG groups, and their baseline and day 15 DOSS scores were assessed. The sample loss rate was conservatively set at 30%. Based on the results and Cohen’s formula, at least 211 participants were required for each group. The formula is as follows:


$$n1=n2=\frac{2\times {\left(Z_{\alpha {/}_{2}}+Z_{\beta }\right)}^{2}\times \sigma^{2}}{{\left(M1-M2\right)}^{2}}÷0.7$$

In this case, the corrected *α* = 0.007, (1−*β*) = 95%. M1 = 4.00, M2 = 5.16, σ =2.39. The effect size was derived from our preliminary randomized trial using FEES-confirmed DOSS scores. Given the lack of prior large-scale rigorous data using gold-standard swallowing assessments, this effect size represents a clinically plausible and realistic improvement supported by our pilot data and clinical experience. This target sample size (n1 = n2 = 211) is higher than that found in related studies retrieved from common databases such as the Web of Science, PUBMED, and CNKI. Sample size calculations based on other parameters yielded results below this value. To ensure the test power, these results were not adopted. The sample sizes in other scenarios are shown in Appendix S5.

### 2.8 Data collection and management

Assessors will be trained in advance in the Dysphagia Research Institute of Zhengzhou University to enhance consistency. In the fourth week, staff will contact participants by phone to remind them to record aspiration symptoms and the close-out timepoint of the study, to facilitate their completion of the follow-up. The database management system of the sponsor center will be used to store data. Only researchers will have access to generated documents and databases. All publicly available content will involve de-identified data. Demographic data will be stored in a separate file, which will only be accessible to the principal investigator after the study ends.

### 2.9 Statistical methods

Generalized Estimating Equations will be used to analyze repeated measures data, including the baseline and outcome DOSS, MSS, YPR-SRS, and PAS. The analyzed effects will include time, between-group, and interaction terms. Significant interaction effects indicate that differential interventions can impact the outcomes over time. Based on the primary outcome, additional analysis will be conducted by comparing the intergroup differences in the proportion of participants who will achieve oral intake at T1, using the chi-square test. The time to improvement of one level from the baseline DOSS and the time to oral intake will be analyzed using stratified log-rank tests, with stratification by the baseline DOSS. Data from patients who do not achieve their goals will be censored at day 42. The number of overt aspiration per person over 6 weeks will be analyzed using negative binomial regression to assess intergroup differences in incidence rates. Kaplan–Meier analysis will be used to handle the occurrence of aspiration pneumonia and to plot survival function curves. Statistical analyses will be based on intention-to-treat, and multiple imputation methods will be used to address partial missing values (31). Furthermore, we plan to perform several sensitivity analyses, including per-protocol analysis using a sample that excludes dropouts, and exclusion of centers from different geographic regions, to explore the stability of the main findings. The significance level *α* has been adjusted to 0.007 based on the number of outcomes (Bonferroni). Statistical analyses will be conducted using Stata v18.0 MP.

### 2.10 Ethical considerations and monitoring

This protocol has been approved by the Ethics Committee of the First Affiliated Hospital of Zhengzhou University (No. 2026-KY-0016), and was registered at ClinicalTrials.gov (NCT07386834, 02/04/2026). This study will adhere to the Declaration of Helsinki. Prior to randomization, all participants must provide written informed consent. Members of the Data Monitoring Committee are independent from the sponsors and funders. They will focus on the incidence, severity, and association of adverse events, and have the right to suggest the termination of the study to the ethics committee for safety reasons.

## 3 Discussion

This study is important as it will rigorously and systematically investigate the effects of oro-esophageal versus nasogastric feeding on swallowing outcomes. This study plans to include a broad sample of inpatients with ischemic stroke, focusing on both general and local swallowing function, and will track functional improvements throughout the intervention period rather than only assessing the baseline and post-intervention status. Our findings are expected to provide evidence-based support for clinical decision-making in enteral nutrition.

Gastrostomy is not widely accepted in East Asia because of its invasive nature. Oro-esophageal tube feeding offers an alternative to nasogastric feeding and is therefore worthy of investigation. Compared with nasogastric feeding, if proven to more effectively improve swallowing function, to be non-inferior in safety, and to help reduce overt aspiration or the risk of aspiration pneumonia, this will provide further support for the promotion of oro-esophageal tube feeding. This will benefit not only clinical practice but also patients’ rehabilitation outcomes and quality of life.

This study will adopt the DOSS as the primary outcome. The consideration for this choice is that the DOSS is a widely accepted, clinically intuitive ordinal scale directly associated with any decision about oral intake and tube weaning. It is routinely used in swallowing rehabilitation and aligned with real-world clinical decision-making.

It is rated based on the gold-standard instrument for dysphagia, ensuring objectivity and physiological grounding. Although the DOSS is ordinal, a one-level improvement over 14 days can represent a clinically significant shift in swallowing function in acute stroke rehabilitation. This change corresponds to reduced aspiration risk, better secretion control, earlier achievement of oral intake, and lower dependence on tube feeding. To address the limited granularity of an ordinal scale, we include the MSS, YPR-SRS, and PAS as secondary outcomes. These measures directly quantify physiological improvements in secretion pooling, pharyngeal residue, and airway protection, which may not be fully captured by DOSS alone. We further strengthen sensitivity by analyzing time to one-level DOSS improvement and time to oral intake, which can capture the speed and timing of recovery rather than only endpoint status.

Due to practical limitations, this study has several limitations. First, a gastrostomy group will not be set, which limits external validity and comparison with global standards. Future comparative studies should consider to explore the differences between oro-esophageal and gastrostomy feeding. Second, this study includes only patients with acute ischemic stroke and severe dysphagia requiring tube feeding in China. Therefore, the findings may not generalize to patients with hemorrhagic stroke, milder dysphagia, other neurological conditions, different healthcare settings, or outside China. Third, given the nature of the intervention, this study lacks blinding of participants, therapists, and some assessors. Additionally, though a summary of the core standardized components has been included, the distinctive intervention protocols of each participating center have not been presented in the Appendix, which may compromise the study’s transparency. Finally, the 14-day intervention period may be insufficient to capture meaningful neuroplastic changes and long-term functional outcomes, despite the inclusion of follow-up measures.
