# Supplementary material for: Effects of oro-esophageal versus nasogastric feeding on dysphagia for ischemic stroke survivors: study protocol for a randomized controlled trial
Source: Front Nutr. 2026 Apr 29;13:1830508. doi: 10.3389/fnut.2026.1830508 (PMC13167509; doi:10.3389/fnut.2026.1830508)
Supplement: Supplementary file 1 [file Table_1.DOCX]

**Effects of Oro-esophageal versus Nasogastric Feeding on Dysphagia for Ischemic Stroke Survivors: Study Protocol for A Randomized Controlled Trial**

Appendices

Appendix S1. Oro-esophageal Tubes


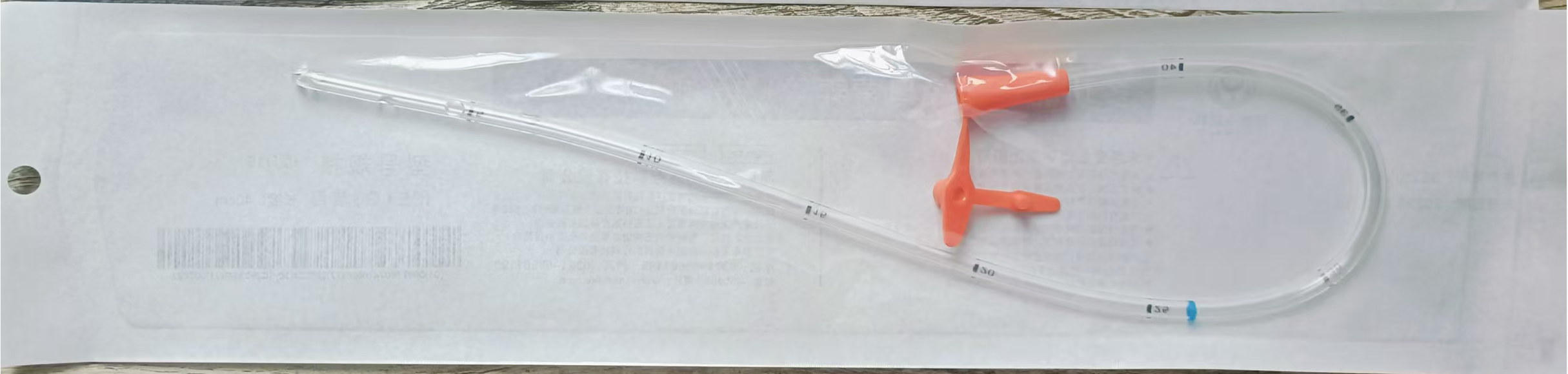


https://baike.baidu.com/item/IOE%E6%8A%80%E6%9C%AF/67242866

Appendix S2. Routine rehabilitation care details

Both groups received routine treatment including health education, guidance, risk factor control, and corresponding medication therapy for secondary prevention. The patients diagnosed with pneumonia underwent corresponding treatment. All centers will implement swallowing rehabilitation training in accordance with the Chinese Guidelines for Rehabilitation Management of Dysphagia (2023 edition). Specifically, the standardized therapeutic components include oral motor training (lip closure, jaw stability, tongue movement, bolus control), pharyngeal facilitation (thermal-tactile stimulation, laryngeal elevation training), swallowing maneuver training (effortful swallow, Mendelsohn maneuver, supraglottic swallow), and secretion management and cough strengthening training. Daily swallowing therapy will be conducted 30–40 minutes per session, once daily, 6 days per week

All therapists follow identical exercise sequences, intensity, and progression rules. All therapists completed pre-study training and obtained certification at the Dysphagia Research Institute of Zhengzhou University.

Regular site visits and remote monitoring are conducted to verify protocol adherence. Any deviation from the unified protocol is documented and corrected promptly. This standardized approach minimizes inter-center, inter-therapist, and inter-treatment variability, ensuring that any observed differences in outcomes can be attributed to the feeding route (oro-esophageal vs. nasogastric) rather than inconsistent swallowing therapy.

Furthermore, device‑based therapies such as transcranial magnetic stimulation, transcutaneous electrical stimulation, music therapy, and Rood technique are provided according to each hospital’s available resources. A standardized protocol was required for each specialized treatment provided by every site hospital, with strict compliance mandated at all times. All protocols were submitted to the Dysphagia Research Institute of Zhengzhou University for review, as well as routine monitoring and follow-up.

Appendix S3. Details in the assessment of time to one-level improvement in the DOSS

To ensure objectivity and consistency, strict operational criteria have been established for determining time to one-level DOSS improvement:

First, Standardized trigger criteria are set: Improvement is suspected only when predefined objective changes are observed, including reduced coughing, improved oral feeding tolerance, decreased secretion pooling, and better laryngeal elevation during training. Specifically, to minimize subjectivity and inter-rater variability, standardized operational trigger criteria are established for identifying potential one-level improvement in DOSS score. Daily evaluations are based on objective, observable signs during routine care and swallowing training.

Trigger criteria for improvement from DOSS 1 to DOSS 2

A participant is considered to have potential improvement if at least one of the following objective changes is observed:

Residuals in the pharynx or oral cavity become manageable with cues or mild efforts

Partial oral intake of one consistency becomes safely tolerable

Volitional cough becomes functionally effective

Swallowing attempts can be consistently initiated

Trigger criteria for improvement from DOSS 2 to DOSS 3

A participant is considered to have potential improvement if at least one of the following objective changes is observed:

Pharyngeal or oral retention is reduced and can be cleared with minimal cues

Bolus loss during the oral phase is decreased

Safe swallowing of one consistency is achieved without constant strategies

Penetration or aspiration is reduced to fewer consistencies

Cough reflex becomes more robust and protective

Only participants who meet the predefined trigger criteria will proceed to confirmatory FEES. The final determination of one-level DOSS improvement is based exclusively on FEES findings, not on clinical observation alone. This hierarchical approach reduces subjectivity while respecting the feasibility of unblinded assessments during the intervention period.

To improve the consistency across assessors, all researchers involve in this assessment attend online meetings to standardize the understanding of the aforementioned content. These meetings are held once every three months after the initiation of the study. The Dysphagia Research Institute of Zhengzhou University provides all sites with a detailed operational manual (available in both electronic and paper versions) containing pictorial examples and decision rules. In addition, participants identified as having the potential for a one-level improvement will have a swallowing screen. This process is required to involve the principal investigator or co-investigator of the respective center. FEES will be performed if and only if the patient, the researcher who identified the potential one-level improvement, and the aforementioned principal or co-investigator reach a consensus.

Additionally, all borderline or ambiguous cases will be reviewed by a central adjudication committee blinded to group assignment to ensure consistent judgment.

Appendix S4. Measures to address potential recall bias and underreporting of aspiration

To address potential recall bias and underreporting of aspiration, the following measures are applied:

Participants or caregivers record aspiration events in a structured paper or electronic log before the last meal of each day. Silent aspiration cannot be identified by self-report. Therefore, silent aspiration is indirectly monitored by tracking new-onset aspiration pneumonia over the 6-week period.

Standard diagnostic criteria for aspiration pneumonia

Aspiration pneumonia is diagnosed if all of the following are present:

New or progressive lung infiltrate on chest imaging

At least one clinical symptom: fever, cough, sputum production, dyspnea, or pleuritic pain

At least one sign: rales or dullness to percussion

No other plausible cause of pneumonia

In addition, sputum culture will be used to identify pathogens when necessary. For complicated or difficult-to-diagnose cases, bronchoscopy may be performed to directly visualize foreign material or obtain deep sputum specimens after obtaining informed consent from the patient.

Appendix S5. Sample sizes in each scenario

In previous studies, no research has reported oro-esophageal versus nasogastric feeding using the Dysphagia Outcome and Severity Scale (DOSS). However, The DOSS and Functional Oral Intake Scale (FOIS) are highly correlated [10.1007/s00455-024-10732-z]. Therefore, the sample size might be estimated based on the FOIS. One study reported the FOIS scores for patients with Wallenberg syndrome using oro-esophageal versus nasogastric feeding [10.1161/STROKEAHA.123.046122]. Based on this, we estimated the sample size using Cohen’s formula, as shown in the table below:

| Standard deviation ($M1$) | Standard deviation ($M2$) | $\sigma$ | α | $Z_{{\alpha/}_{2}}$ | 1-β | $Z_{\beta}$ | Dropout rate | **Result (each arm)** |
| --- | --- | --- | --- | --- | --- | --- | --- | --- |
| 2.121 | 3.283 | 1.259 | 0.05 | 1.96 | 0.80 | 0.842 | 0.3 | **27** |
| 2.121 | 3.283 | 1.259 | 0.05 | 1.96 | 0.90 | 1.282 | 0.3 | **36** |
| 2.121 | 3.283 | 1.259 | 0.05 | 1.96 | 0.95 | 1.645 | 0.3 | **43** |
| 2.121 | 3.283 | 1.259 | 0.007 | 2.701 | 0.80 | 0.842 | 0.3 | **42** |
| 2.121 | 3.283 | 1.259 | 0.007 | 2.701 | 0.90 | 1.282 | 0.3 | **54** |
| 2.121 | 3.283 | 1.259 | 0.007 | 2.701 | 0.95 | 1.645 | 0.3 | **64** |

All results were <211 and were not directly based on the DOSS. Therefore, none were adopted.

In addition, we also performed the estimation based on the pooled odds ratio (OR) from a meta-analysis. This study included several small-scale randomized controlled trials, most of which were not sufficiently rigorous in quality and adopted swallowing screening instead of formal assessment [10.21037/apm-21-736]. The authors reported that oro-esophageal tube feeding could significantly increase the rate of improvement in dysphagia (odd ratio [OR] = 5.22, 95% confidence interval [CI]: 3.38–8.07). Based on this, we estimated the sample size, as shown in the table below:

| OR | P_0_ | α | $Z_{{\alpha/}_{2}}$ | 1-β | $Z_{\beta}$ | Dropout rate | **Result (each arm)** |
| --- | --- | --- | --- | --- | --- | --- | --- |
| 5.22 | 0.1 | 0.007 | 2.701 | 0.95 | 1.645 | 0.3 | **55** |
| 5.22 | 0.2 | 0.007 | 2.701 | 0.95 | 1.645 | 0.3 | **46** |
| 5.22 | 0.3 | 0.007 | 2.701 | 0.95 | 1.645 | 0.3 | **39** |

All results were <211 and were not directly based on the DOSS. Therefore, none were adopted.

Finally, we estimated the sample size using a preliminary study, as shown in the table below. Finally, we selected the scenario with the **largest sample size (n1=n2=211)** to minimize potential bias as much as possible.

| Standard deviation ($M1$) | Standard deviation ($M2$) | $\sigma$ | α | $Z_{{\alpha/}_{2}}$ | 1-β | $Z_{\beta}$ | Dropout rate | **Result (each arm)** |
| --- | --- | --- | --- | --- | --- | --- | --- | --- |
| 4 | 5.16 | 2.388 | 0.05 | 1.96 | 0.80 | 0.842 | 0.3 | **88** |
| 4 | 5.16 | 2.388 | 0.05 | 1.96 | 0.90 | 1.282 | 0.3 | **118** |
| 4 | 5.16 | 2.388 | 0.05 | 1.96 | 0.95 | 1.645 | 0.3 | **146** |
| 4 | 5.16 | 2.388 | 0.007 | 2.701 | 0.80 | 0.842 | 0.3 | **140** |
| 4 | 5.16 | 2.388 | 0.007 | 2.701 | 0.90 | 1.282 | 0.3 | **178** |
| 4 | 5.16 | 2.388 | 0.007 | 2.701 | 0.95 | 1.645 | 0.3 | **211** |

We performed several sensitivity analyses, including excluding the first, middle, and last thirds of the preliminary study data, respectively. The results showed that the single-arm 211 design using the full dataset was the most robust.

| Standard deviation ($M1$) | Standard deviation ($M2$) | $\sigma$ | α | $Z_{{\alpha/}_{2}}$ | 1-β | $Z_{\beta}$ | Dropout rate | **Result (each arm)** |
| --- | --- | --- | --- | --- | --- | --- | --- | --- |
| 4.78 | 5.94 | 2.083 | 0.007 | 2.701 | 0.95 | 1.645 | 0.3 | **161** |
| 2.9 | 4.1 | 2.355 | 0.007 | 2.701 | 0.95 | 1.645 | 0.3 | **195** |
| 3.85 | 5.05 | 2.417 | 0.007 | 2.701 | 0.95 | 1.645 | 0.3 | **204** |
